# Optimization algorithm of CT image edge segmentation using improved convolution neural network

**DOI:** 10.1371/journal.pone.0265338

**Published:** 2022-06-03

**Authors:** Xiaojuan Wang, Yuntao Wei

**Affiliations:** College of Electronic Information Technology, Jiamusi University, Jiamusi, China; Sri Eshwar College of Engineering, INDIA

## Abstract

To address the problem of high failure rate and low accuracy in computed tomography (CT) image edge segmentation, we proposed a CT sequence image edge segmentation optimization algorithm using improved convolution neural network. Firstly, the pattern clustering algorithm is applied to cluster the pixels with relationship in the CT sequence image space to extract the edge information of the real CT image; secondly, Euclidean distance is used to calculate similarity and measure similarity, according to the measurement results, convolution neural network (CNN) hierarchical optimization is carried out to improve the convergence ability of CNN; finally, the pixel classification of CT sequence images is carried out, and the edge segmentation of CT sequence images is optimized according to the classification results. The results show that the overall recognition rate of this method is at a high level. The training time is obviously reduced when the training times exceed 12 times, the recall rate is always about 90%, and the accuracy of image segmentation is high, which solves the problem of large failure rate and low accuracy.

## 1. Introduction

People pay more and more attention to the living environment and health, especially health problems [1, 2]. Medical imaging can provide patients with more intuitive, clearer and more accurate diagnosis with higher accuracy [3]. With the continuous development of medical imaging technology, doctors’ diagnosis and treatment methods are becoming more and more important [4, 5]. Due to the wide application of new medical digital imaging technologies such as Magnetic Resonance Imaging (MRI), Computed Tomography(CT) and ultrasonic(Us), high-resolution medical image data can be obtained. However, reasonable and effective use of these valuable medical image data is the key to help doctors diagnose and treat. Computed tomography sequence image has the advantages of real-time, non-destructive, low cost, etc., and has been widely used in disease prevention, diagnosis and treatment. Computed tomography image processing methods have become one of the main directions of blood lipid at home and abroad [6, 7]. Deep learning is widely used in medical image processing, represented by convolutional neural networks, it has developed rapidly in recent years [8, 9]. Through the establishment of multiple hidden layer neural networks and a large amount of data training, more useful feature information can be extracted from the data, so that medical images can be processed more accurately, and better results have been obtained for computed tomography image processing [10, 11].

Literature [12] proposed image segmentation processing based on convolutional neural network, the neural network is trained by data multi-scale feature fusion and residual connection, the convolution neural network segmentation model is optimized, and the image with high definition is segmented by spline interpolation; Literature [13] combined residual learning and densely connected network to fully extract image features, and added hollow convolution to the neural network. The improved neural network has higher accuracy and sensitivity in processing images; Literature [14] fused image feature information, according to the improved convolution neural network, the improved network is used to roughly segment the image, and a batch regularization layer is added after each convolution layer to speed up the convergence and improve the segmentation accuracy.

In addition, due to the poor visibility of computed tomography sequence images and easy to observe changes, it is impossible to determine the size and shape of relevant organs in most clinical practice. At the same time, manual segmentation is mainly used in clinic. However, the repeatability of manual segmentation is poor and time-consuming. It largely depends on the experience and ability of doctors. The edge segmentation results of different doctors are different, and the edge segmentation results of the same doctor at different times will be slightly different, that is, the repeatability is poor. Real time edge segmentation of computed tomography sequence images is an important technology in clinical applications such as brachytherapy. In previous studies, only computed tomography images were processed without considering their sequence attributes. The main contributions of this paper are as follows: (1) In this paper, the pixels with certain relationship in computed tomography sequence image space are clustered to obtain the edge information of computed tomography sequence image and de-noise it at the same time. (2) In order to solve the gradient disappearance problem of low-level neural network during back propagation. In this process, we not only consider the particularity of computed tomography sequence images, but also improve the convergence ability of convolution neural network.(3) The details of the target and the corresponding spatial dimensions are gradually recovered through network layers such as de-convolution layers. At the same time, hollow convolution can increase the receptive field and keep the weight parameters unchanged, which can effectively maintain the resolution of computed tomography sequence images.

The organization of this paper is as follows. We introduce the introduction and related works in Section1 and Section2. Then, we describe methodology in Section3. We further present the experimental results and discussions in Section4. Finally, we conclude the paper in Section 5.

## 2. Related work

For computed tomography image segmentation related issues, many studies have also appeared. The image segmentation method proposed by Literature [15] was based on a large number of prior segmentation methods. These methods are based on liver regions that are imprecise or require a lot of training. Literature [16] segmented and marked each cell in the bright-field image according to the morphological features of yeast cells to identify the seed points on the cell contour. The edge of yeast cells was successfully extracted from the light field image of sparsely distributed cells. The results show that the dense cell images can be segmented and marked correctly. Literature [17] improved the contrast between normal liver parenchyma and tumor tissue by adaptive piecewise nonlinear enhancement and iterative convolution operation. On this basis, the enhancement result and image boundary information are effectively integrated into the graph cut energy function, false segmentation regions are removed, and the initial segmentation results are optimized. This realizes the preliminary automatic segmentation of liver tumors, and realizes effective automatic segmentation of liver tumors in the computed tomography sequence. Literature [18] proposed a high-precision region segmentation method to constrain the continuous features of computed tomography images. This method takes the updated mean value of seed points and continuous segmentation as constraints, realizes the high-precision region segmentation of computed tomography image features, and reduces the possibility of holes and non-vascular information in the process of liver segmentation. In foreign research, Literature [19] proposed a new method for measuring voxel porosity and permeability of gray areas. The lattice evaluation method was used to evaluate the permeability of the reservoir, and computed tomography images were obtained. After obtaining the pore size distribution curve through experiments, according to the image gray scale, the integral pore volume, and the linear relationship between the computed tomography number and the porosity of the single element, the boundary condition constraints of image segmentation are increased. Literature [20] proposed a feature segmentation method based on the fuzzy edge of the image. After preprocessing, the impurities and noise in the image are removed, and the processed image is segmented by the fuzzy edge. Experiments show that this method can effectively improve the efficiency and accuracy of segmentation, and shorten the segmentation time of fuzzy edges.

On the basis of existing research, only computed tomography images were processed, and their sequence attributes were not considered. This paper proposed a computed tomography sequence image edge segmentation optimization algorithm based on improved convolutional neural network. Experiments show that the overall recognition rate of this method is high, the error rate is low, the recall rate is always around 90%, and the accuracy of image segmentation is much higher than other literature algorithms, which can be as high as 0.99, which has certain advantages.

## 3. Methodology

### 3.1 Integration of edge information of computed tomography sequence image

In order to obtain the edge information of the computed tomography sequence image, it is necessary to remove the edge information under the influence of noise. The images that meet the requirements are divided into different types by a simple pattern clustering method, and the edge information of the real image is extracted as much as possible [21]. Let automatic threshold be *f*, and the intersection *n* is obtained by intercepting multiple fitting curves in the data space. This intersection is the edge point of the image, and due to the threshold *f*_0_, the noise edge point is not intercepted and eliminated. At this time, the information at *n* is

$$A_{G}=f_{L}f(n)-f_{H}f0$$ (1)

where *A*_*G*_ refers to image edge point information function at intersection *n*. Therefore, we can get the actual image edge point cluster set:

$$A^{\prime }=A_{G}\overset{v}{\underset{n=1}{\sum }}k_{n}+k_{0}$$ (2)

where *k*_0_ refers to initial cluster information, *k*_*n*_ refers to final cluster information. By solving this equation, the position of the image edge point *n* can be obtained as follows:

$$A_{n}=2A^{\prime }(f_{L}+f_{H}){\left(1-A_{e}\right)}^{e-j}$$ (3)

where *A*_*e*_ refers to operation unit position of the number of clustering information, *e* and *j* are cluster dimensions. At this time, the edge points *n* of the computed tomography sequence image are detected in turn, and the noise edge points are eliminated in turn [22].

Because different features of computed tomography sequence image come from different feature extraction methods, they have different properties. For example, different machine parameters, different date range, different patients, etc., which require us to calculate the computed tomography sequence image super-pixel similarity, we should form an independent calculation similarity for different practical application properties [23, 24]. Therefore, if you want to process all different computed tomography sequence images at the same time, and the computed tomography sequence image features are different, then all the image features are combined to form a high-dimensional feature vector *A*_*v*_, and then use the Euclidean distance to calculate the similarity *h*_*p*_:

$$h_{p}=A_{n}\sum_{i=0}^{v}\sum_{j=0}^{e}{\left(1-A_{v}\right)}^{j-i}$$ (4)

The super-pixel similarity *h*_*k*_ of computed tomography sequence image features is:

$$h_{k}=h_{p}\sum_{j=0}^{e}F_{i,j}A_{v}^{i}$$ (5)

where *F*_*i*_ refers to similarity measure. After normalization, the super-pixel similarity of computed tomography sequence image features will dominate the similarity measure, which weakens the low-dimensional similarity characteristics of super-pixels. Make different features have different weights *m* to balance the proportion of different features in the similarity calculation [25, 26] and reduce the proportion of irrelevant features *G*_*H*_:

$$G_{H}=m\sum_{n=1}^{v}k_{n}h_{k}{\left(1-A_{e}\right)}^{e-j}$$ (6)

The proportion *G*_*H*_ of each detected image edge point is connected into a curve to obtain continuous image edge information.

### 3.2 Edge segmentation of computed tomography sequence image based on improved convolutional neural network

#### 3.2.1. Analysis of convolution neural network improvement

The edge information of the computed tomography sequence image can be extracted using the above process [27, 28]. When the distribution of the input value gradually shifts or changes, the entire distribution gradually approaches the upper and lower ends of the value range of the nonlinear function, resulting in the disappearance of the gradient of low-level neural network in the back-propagation process, which is the fundamental reason for the slow convergence speed of deep-seated neural network training [29, 30]. Convolution neural network has the ability to quickly converge, and can promote the edge segmentation of computed tomography images. According to the particularity of computed tomography sequence images, convolution neural network layering optimization is carried out. The optimization results are as follows:

1. Input layer: According to different recognition objects, computed tomography images of specific size are input. This selects 28 * 28 and 50 * 50 medical images;
2. Convolutional layer C1: The input image is convolved with six 5×5 convolution kernels to form six 24×24 feature maps, which are obtained by convolution operation (28–5+1 = 24)
3. The down-sampling layer S1: Each feature scale of the C1 layer is scaled to 2, ensuring that six 12*12 down-sampling feature maps are formed without repeated down-sampling;
4. Convolutional layer C2: Convolve sixteen 5×5 convolution kernels to form sixteen 8×8 (12–5+1 = 8) feature maps;
5. The down-sampling layer S2 is composed of sixteen 4×4 feature maps, and each neuron is connected to the 2×2 neighborhood of the C1 layer;
6. Fully connected layer F1: Each feature map is connected with all feature maps of S2 layer, and the feature map of S2 layer is connected into one-dimensional input vector;
7. Output layer: The output is divided into two categories: edge segmentation success and sigmoid function

**Fig 1:**
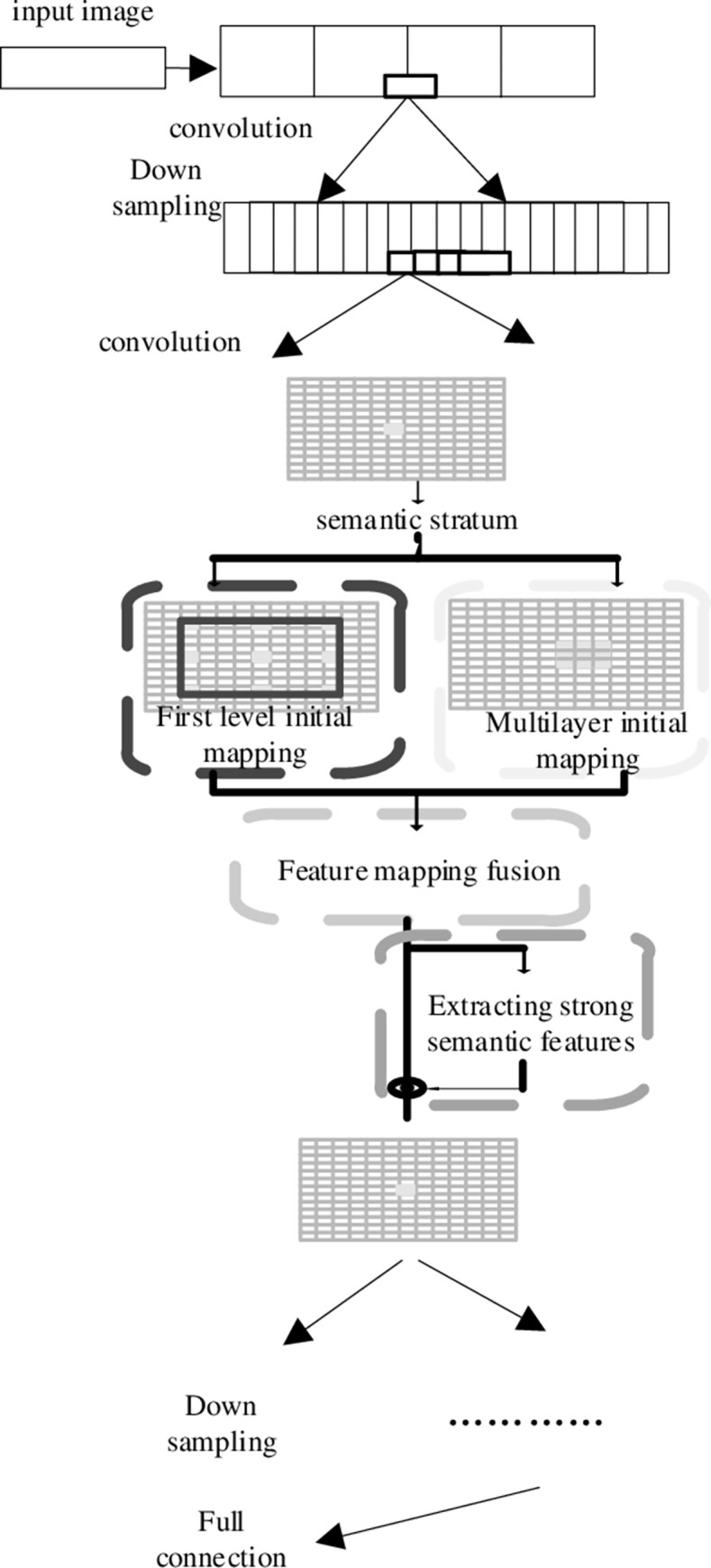
Convolution neural network edge segmentation process of computed tomography image sequence.

In the edge segmentation of the computed tomography sequence image, each pixel has corresponding case identification number information and semantic category information such as department and disease, the lesion feature information is more important, which can be predicted by actual branches and semantic branches [31, 32]. Taking instance branching as an example, the existing network framework is designed according to the evaluation index of instance edge segmentation, that is, only the prediction accuracy of each instance is considered, and the relationship between different types of instances is not processed. This method is difficult to determine the overlapping areas of different types of instances. In order to solve this problem, a new multi-scale network structure is adopted, which can be modified directly on the original network framework. This structure can be divided into two parts: (1) The bottom-up path is used as the original basic network, and the last layer feature map of each stage is used as the feature map to be fused. The feature map of each resolution is introduced into the feature map at twice the resolution, and each resolution feature map is superimposed pixel by pixel; (2) The top-down path corresponds to the upper sampling of the feature map. The high-resolution feature mapping is obtained by up sampling, so that the high-resolution feature mapping has a higher level of semantic information. It is shown in Fig 1.

It is seen from Fig 1 that the feature maps used for prediction of each layer are fused into feature maps with different resolutions and semantic levels, and feature maps with different resolutions are fused into corresponding resolutions. This method not only guarantees the resolution of each layer, but also guarantees strong semantic features. It not only adds cross-layer connections to the original basic network, but also has low requirements for calculation time and speed, which improves the convergence speed.

#### 3.2.2. Computed tomography sequence image edge segmentation optimization algorithm

Generally, the convolution neural network edge segmentation algorithm is to classify the input image. However, the computed tomography sequence image has sequence attributes, and the amount of calculation during classification is large, and the best single resolution cannot be guaranteed. Therefore, this method is adopted. Based on the coding and decoding, this paper proposed a practical deep fully convolutional neural network. A low-resolution encoder transforms the image to the full input-resolution feature map to perform pixel classification of computed tomography sequence images, optimize edge segmentation, and the algorithm is as follows:

Input: Computed tomography sequence image original pixels and cluster information;

Output: Computed tomography sequence image edge segmentation result.

**Fig 2:**
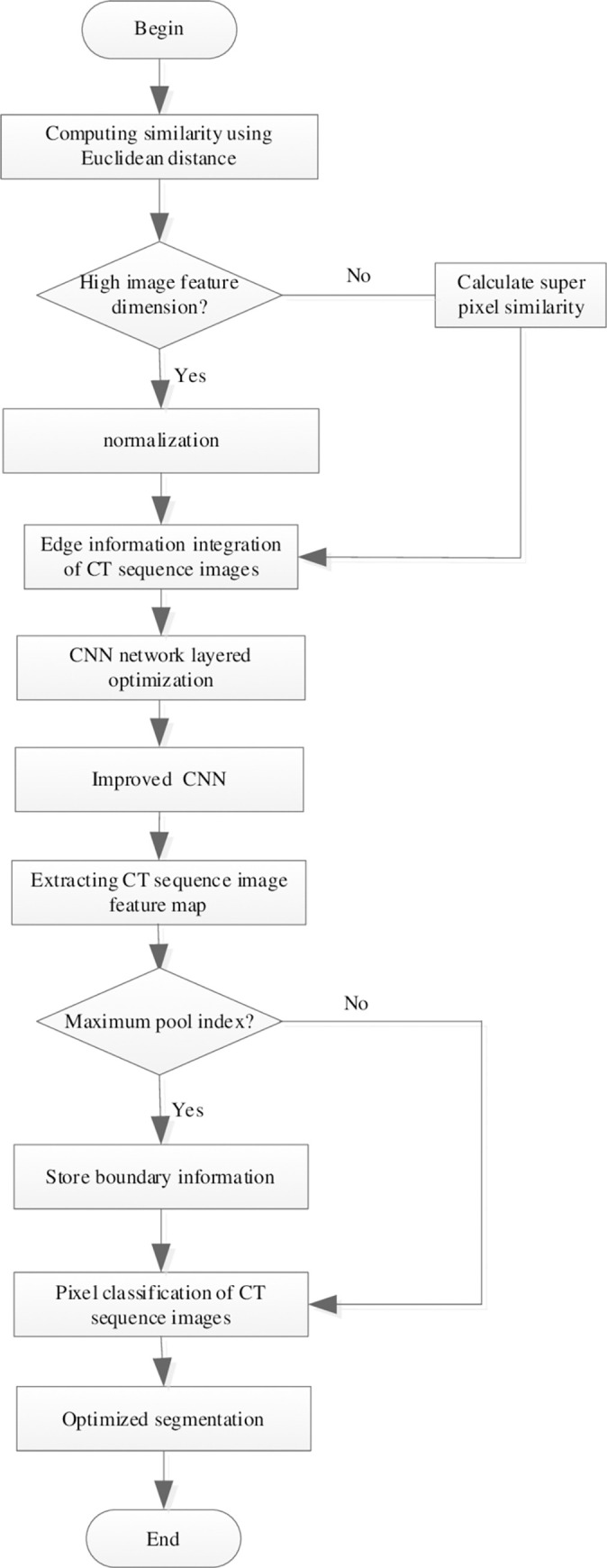
Edge optimization segmentation process of computed tomography sequence image.

Initialize the computed tomography sequence image information and the convolutional neural network, and use the improved convolutional neural network to optimize the image edge segmentation. The steps are as follows:

1. The decoder uses its low resolution *s* input characteristic map and the pool index calculated by the maximum pool steps *S*_*mj*_ of the corresponding encoder for nonlinear up sampling. The sampled feature vector can be expressed as:
$$E(A)=\sum_{j=1}^{v}\frac{S_{1j}+S_{2j}+\ldots +S_{mj}}{s}$$ (7)
  Since the up-sampled feature map is sparse, then *E*(*A*) is convolved with a trainable filter to generate a dense feature map:
$$E{\left(A\right)}^{\prime }=\frac{n\times S_{packet}}{n\times S_{packet}+1}+E(A)$$ (8)
2. Due to the superposition of maximum pooling and sub-sampling $x_{k}^{'}$, the boundary detail loss coefficient *C*_*i*_ increases, and boundary information can be captured and stored in the encoded feature map. At this time, the border information $P(x_{1}^{'}|C_{i})$ can be shown as:
$$P(x_{1}^{'}|C_{i})=\prod_{k=1}^{q}E{\left(A\right)}^{\prime }P(x_{k}^{'})C_{i}S_{packet}$$ (9)
3. In order to improve efficiency, only the boundary information of the largest pool index is stored here. For each 2x2 pool window, the step size is 2 in principle. The storage efficiency is higher than the storage feature map with floating precision. It is worth noting that the final decoder generates a multi-channel feature map and sends the input information to the classifier to obtain the pixels of the multi-channel image. Each pixel is classified independently, and the classification function is:
$$Sim(A,B)=\frac{2I_{3}}{I_{1}+I_{2}}$$ (10)
4. The predicted edge segmentation has the largest probability for each pixel, that is, the class with the largest probability. However, since the step size is reduced, the maximum probability class *x*_max_(*i*) is expressed as:
$$x_{\max}(i)=r(i,j)P(x_{1}^{'}|C_{i})$$ (11)
  where (*i*,*j*) is the pixel coordinates.
5. In this way, we can get a denser feature map. However, it also raises another question: the change of perception space. It is felt that the field is directly related to the step length, that is, according to Formula 11. If the step size becomes smaller and the sensory field does not change, the convolution kernel should be increased. Therefore, the last sampling operation below the maximum pool layer will be removed, and the filter will be up-sampled. The sampling matrix is:
$$M=[\frac{\Delta_{\max}^{m}-\Delta_{\min}^{m}}{G_{H}\cdot x_{\max}(i)}]$$ (12)
  where $\Delta_{\max}^{m}$ and $\Delta_{\min}^{m}$ represent the maximum and minimum convolution kernels, respectively.
6. When the resolution of the down-sampling feature image decreases, the hole convolution is inserted between the continuous filter values to increase the receptive field in the convolution process, which can effectively improve the resolution of the feature map and improve the image quality. The hollow convolution function is:
$$\mu_{x}=\frac{M}{N}[i+r^{2}k/w_{k}]$$ (13)
  In the final feature aggregation layer, all the fully connected layers are replaced by hollow convolution. *N* refers to output features, *r* refers to void factor. *w*_*k*_ is the k-th parameter of the convolution kernel.
  The hollow convolution with different coefficients is used to stack to obtain a larger receptive field, which can obtain richer multi-scale image feature information in the encoder stage. It can not only better identify the lesion area of edge segmentation, but also obtain a smooth edge contour, so as to realize the optimization algorithm design of computed tomography sequence image edge segmentation.
7. End.
  Based on the above algorithm process, the optimal edge segmentation of computed tomography sequence images can be realized, as shown in Fig 2.

## 4. Experiments and results

### 4.1. Experimental environment

The software and hardware environment involved in this experiment are as follows: Hardware environment: hard disk is 3.5tb, memory is 32.0gb, Intel Xeon CPU is e5-2407 v2@2.40GHz. Software environment used Windows 7 operating system and MATLAB R2014b.

### 4.2. Data sets

The data source of this experiment is MURA data set and computed tomography data set of imaging department of a hospital. MURA data set: released by Stanford, one of the largest public radiographic image data sets. In this experiment, t chest x-ray diagnosis of radiation pneumonia images was selected as one of the data sources, and a total of 5000 lung computed tomography images were selected. Computed tomography data set of imaging department of a hospital: The data of 5000 cases of lung in situ computed tomography images in the imaging department of a municipal hospital were collected.

### 4.3. Experimental steps

1. In order to avoid over fitting caused by too small data sets, lung tumors are not limited to a specific type of lung tumors, and only the image data marked by experts as lung cancer are used as experimental data. The experimental data includes images of 200 lung tumors images and 800 normal lungs images.
2. Image preprocessing: the false color in the computed tomography image was removed, the local features of the three modal images in the region of interest area was extracted, and it was normalized to 28 * 28 and 50 * 50 experimental images.
3. Construct different sample spaces: Since the three modes of computed tomography, and positron emission tomography / computed tomography correspond to different image data of the same patient, computed tomography, positron emission tomography and positron emission tomography / computed tomography are constructed through pre-extraction, pre-processing, convolution neural network training and testing of region of interest area. Three different sample spaces for computed tomography.
4. Single convolution neural network structure: According to the given method, an improved convolution neural network model is constructed by using the parameter transfer method, and its performance is compared comprehensively. The effectiveness and superiority of this method are verified by studying the influence of the number of iterations and batch information on the recognition efficiency and training time of the convolutional neural network model. By studying the effects of iteration times and batch information on the recognition efficiency and training time of convolutional neural network model, the effectiveness and superiority of this method are verified.

### 4.4. Experimental indicators

1. The relationship between the number of iterations, recognition rate and training time: In the convolution neural network iteration layer, when a series of operation steps are judged to be repeated, the accuracy of the latter is obtained by the former in turn. Each result of this process can be obtained by performing the same operation steps on the previous results. Therefore, its calculation formula is:
$$u_{n}=(u_{n}-1)\times 2\;(n\geq 2)$$ (14)
  where *u*_*n*_ is the execution amount of repeated instruction *n*.
2. Misclassification rate: Taking the influence of the amount of information of batch computed tomography image application on the recognition rate and training time as an example, to determine the relationship between the number of training *pS* and the training time *T*_*K*_, the judgment formula is:
$$T_{P}=T_{K}(T_{q}/T_{P})+p_{S}$$ (15)
  where *T_q_* refers to the times to identify errors, and *T*_*P*_ refers to total identification times.
3. Recall rate: When segmenting, set as much multi-scale image feature information as + as possible. The larger the ratio of the amount of retrieved relevant information to the total amount of relevant information in the system, the more +, the higher the recall rate high.
4. CT sequence image edge information integration effect: In the computed tomography sequence image edge information integration, the key point is to accurately calculate the edge point position of the image *A*_*e*_. The edge information integration effect of CT sequence images in this paper is verified by comparing the position *A*_*e*_ of image edge points calculated by Formula 3 with the proportion of actual image edge points.
5. Image segmentation accuracy: The accuracy of image segmentation is an indicator. The performance of this algorithm is verified by comparing this algorithm with other algorithms. The accuracy calculation formula is as follows:
$$\mathrm{Segmentation}\;\mathrm{accuracy}=\frac{J_{i}}{J}\times 100\%$$ (16)
  where *J*_*i*_ refers to calculated image segmentation result, and *J* refers to actual image segmentation result.

### 4.5. Results and discussion

**Fig 3:**
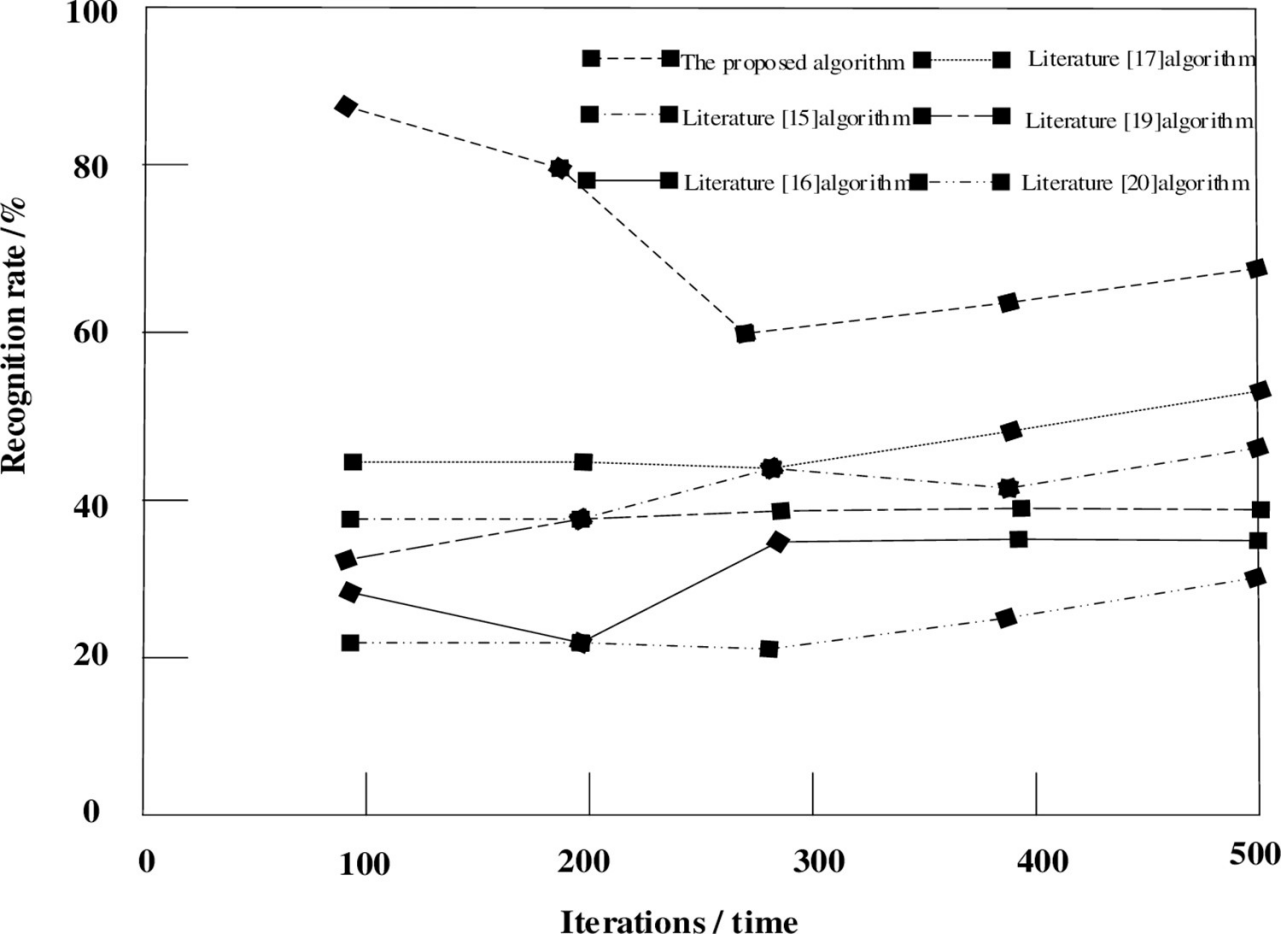
Relationship curve between iteration times and recognition rate.

In order to ensure the unity of the data itself and to better verify the overall performance of the improved convolutional neural network, the relationship between the number of iterations and the recognition rate in the improved convolutional neural network model is discussed. It is shown in Fig 3.

According to Fig 3. In the process of iteratively extracting the edge information of the original image, when the number of iterations increases, the recognition rate rises slightly. The method shows a downward trend in the number of iterations of 100–300, and an upward trend before and after 300–500 iterations. However, the overall recognition rate is higher than the other five algorithms. This shows that this method can simultaneously extract the detailed features and higher-level features of the original image. This is because traditional image recognition methods are based on the region of interest of the entire image, first perform edge segmentation, then extract features, and use a suitable classifier for recognition. This tedious process is simplified by the convolution and down-sampling operations of the convolution neural network, and is directly completed by the convolution and down-sampling operations of the convolution neural network.

**Fig 4:**
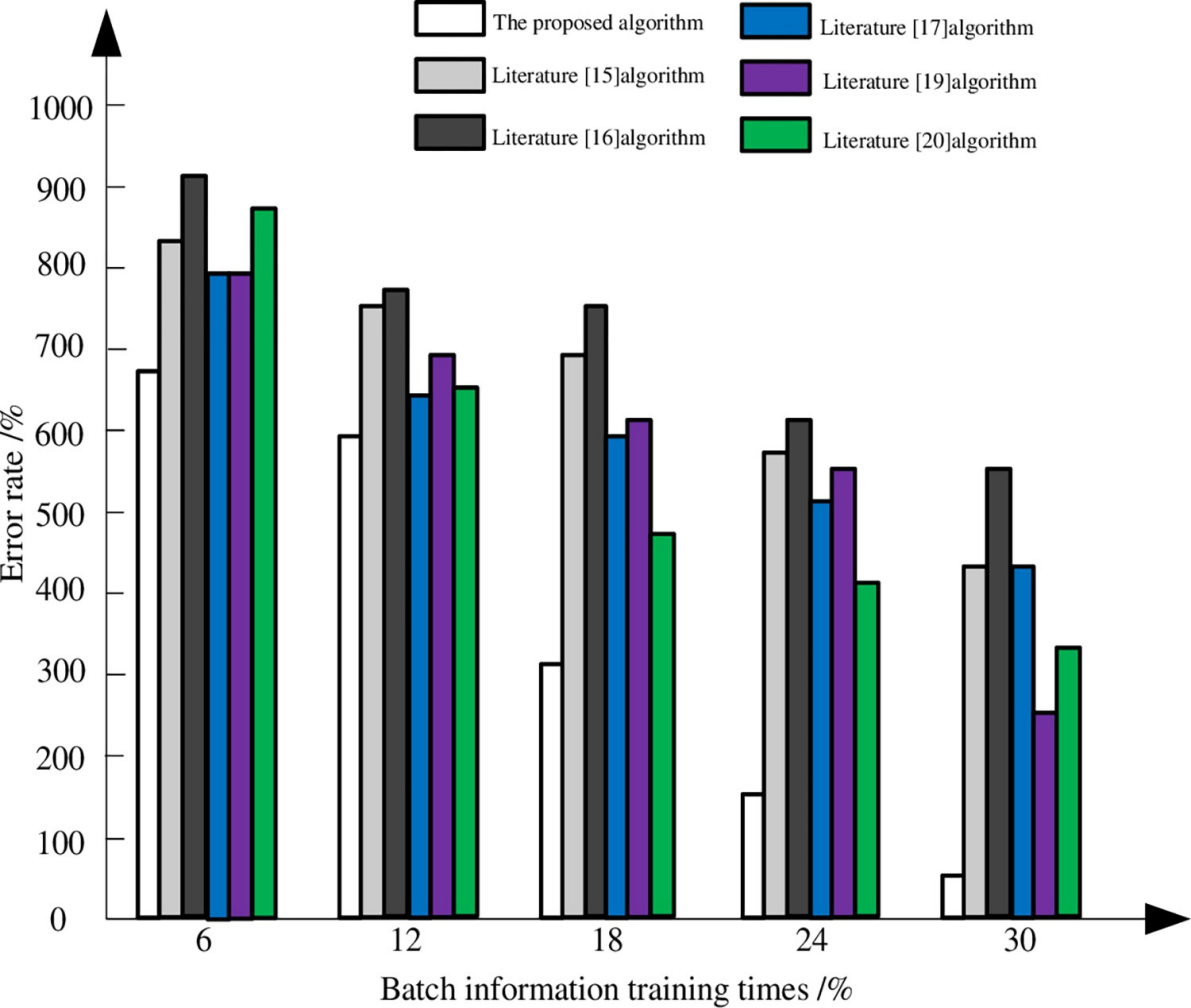
Comparison results of error rate of different methods.

Since the omnidirectional boundary segmentation of lung images needs to acquire and learn the basic information of lung images, so as to achieve the purpose of accurate recognition, the ability of different methods to obtain original lung image information is compared. Taking the results of batch information acquisition as an example, the false detection rates of the five algorithms are compared. It is shown in Fig 4.

According to Fig 4. Regardless of the number of training times, the error rate of the five methods decreases with the increase of the number of training times. When the number of training times exceeds 12, the error rate of the algorithm in this paper drops sharply and the recognition rate increases. And as the number of training continues to increase, the recognition rate remains at a high level, and there is basically no training. The training effect of other methods is not ideal, especially when the number of training is 12. The error rate of Literature [15], Literature [16] and Literature [17] algorithms still remain at a high level, and there is no significant drop. The error rate of Literature [19] and Literature [20] algorithms has decreased. However, it is still much higher than the method in this paper. The reason for this difference is that the method in this paper optimizes the convolution neural network hierarchically and improves the convergence of the convolution neural network. This can train a large amount of data, reduces training time, has a higher recognition rate, and also reduces training time.

**Fig 5:**
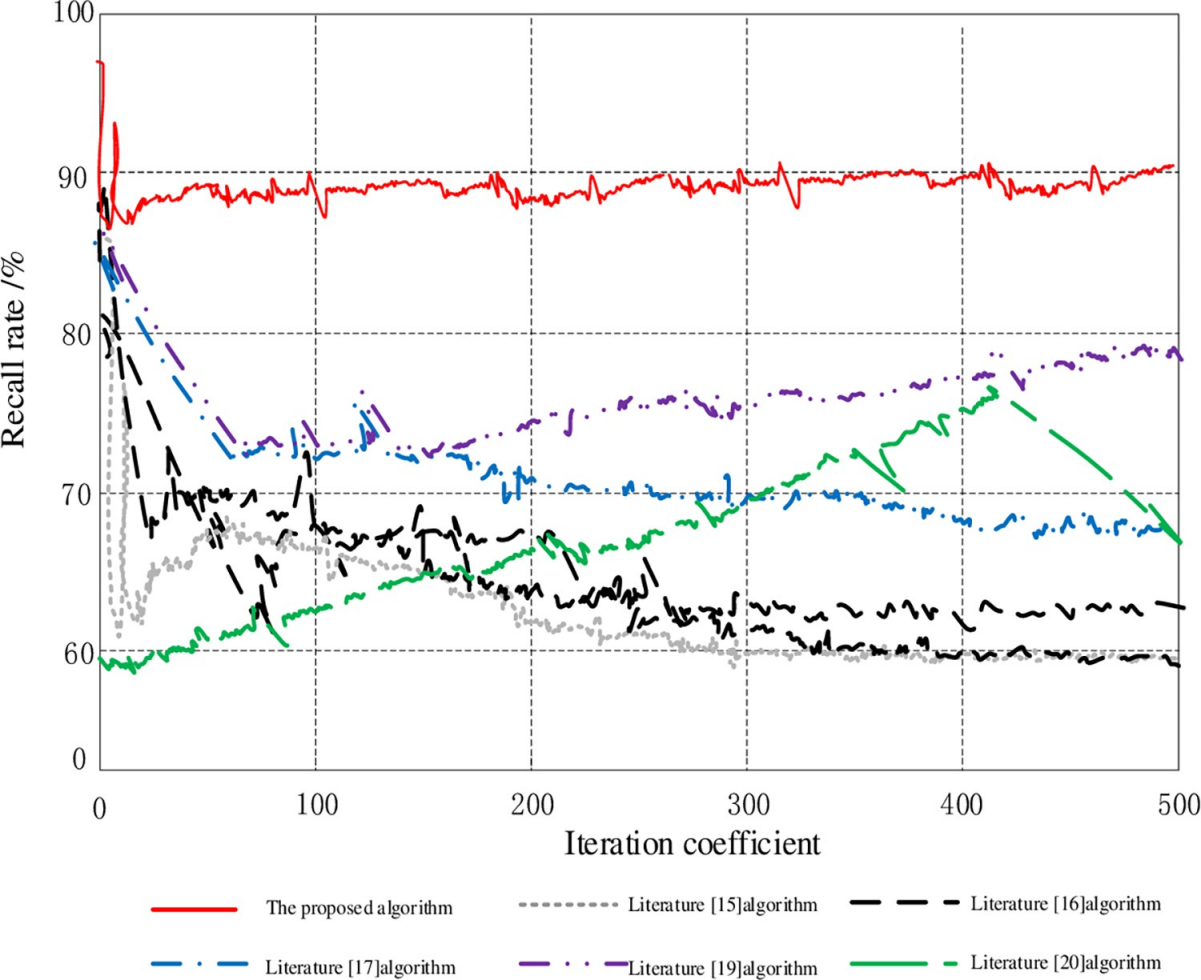
Comparison of recall results.

In the process of computed tomography image segmentation, this paper obtains richer multi-scale image feature information in the encoder stage. The recall of feature information reflects the richness of information acquisition of the segmented image in the process of extracting features, analyzes the relationship between recall curves of different methods, and compares the results of recall. It is shown in Fig 5.

According to Fig 5, it can be seen that the recall curve of the method in this paper is higher than that of other literature methods, and it has a higher advantage. The recall rate is always around 90%, while Literature [15], Literature [16], Literature [17] and Literature [19] algorithm, the recall rate decreases with the increase of the iteration coefficient. Although the Literature [20] algorithm increases its recall rate with the increase of the iteration coefficient, the highest is about 75%. It can be seen that the recall rate of this method is higher than other methods. This is because the hollow convolution is inserted between successive filter values, which increases the receptive field during the convolution process, which can effectively improve the resolution of the feature map. In the encoder stage, richer multi-scale image feature information can be obtained, and the initial recall rate is increased. When the scale of the image feature information remains unchanged, the overall recall rate increases.

**Fig 6:**
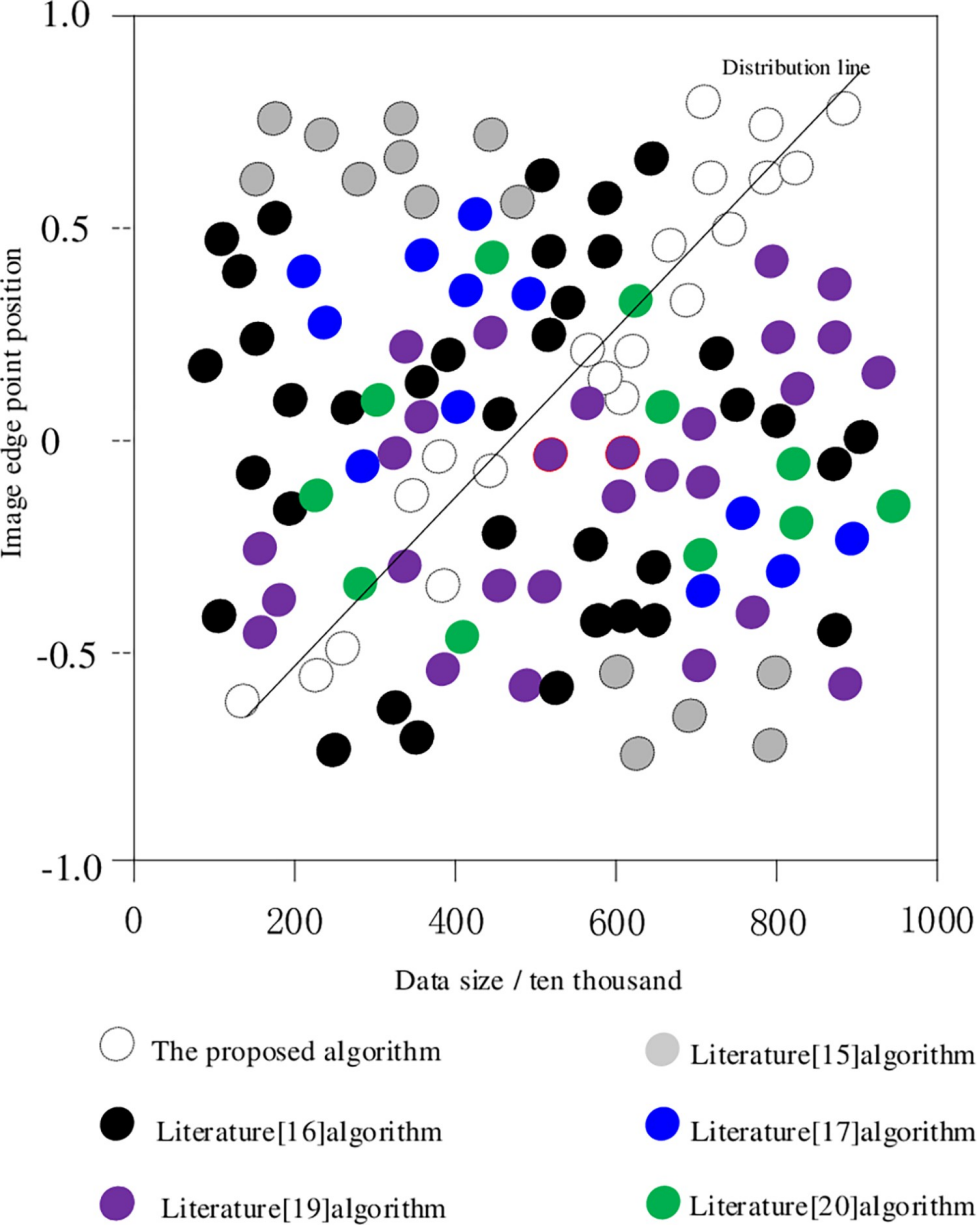
Comparison of distribution of image edge points.

1. The comparison results of image segmentation accuracy in different literature are shown in Fig 6.

According to Fig 6 the image edge points of the algorithm in this paper are tightly distributed on both sides of the distribution line, and the distribution is relatively uniform, which is in good agreement with the actual distribution of the image edge. Other Literature algorithms are scattered and far away from the distribution line, and the distribution is scattered and uneven. This is because the algorithm in this paper uses the threshold *f*_*0*_ limit, and the noise edge points are not intercepted to be eliminated, thereby reducing the interference of the noise edge points, and the distribution result is closer to reality.

The comparison results of image segmentation accuracy in different literatures are shown in Table 1.

**Table 1:** Comparison of image segmentation accuracy.

Data size / hundred	The proposed algorithm	Literature [15] algorithm	Literature [16] algorithm	Literature [17] algorithm	Literature [19] algorithm	Literature [20] algorithm
200	0.95	0.60	0.53	0.72	0.52	0.36
400	0.96	0.63	0.54	0.72	0.55	0.50
600	0.97	0.62	0.53	0.76	0.51	0.42
800	0.98	0.68	0.58	0.73	0.58	0.43
1000	0.99	0.69	0.55	0.75	0.59	0.45

By analyzing the results in Table 1, it can be seen that the image segmentation accuracy of the Literature [20] algorithm is the lowest, Not more than 0.50, followed by the Literature [16] and Literature [19] algorithms, and the image segmentation accuracy is below 0.60. Literature [15] and Literature [17] have relatively high accuracy rates, especially Literature [6] can reach up to 0.76, while the segmentation accuracy of this method is between 0.95–0.99. It can be seen that under different data size conditions, the segmentation accuracy of other Literature algorithms is far below the method in this paper. Therefore, it can be explained that the method in this paper has a better operation effect on capturing and storing boundary information and the operation effect of storing boundary information is good, which greatly improves the retention effect of image boundary information, and then improves the accuracy of image segmentation.

## 5 Conclusions

Computed Tomography sequence image is currently an important method for detecting many diseases. The accuracy of computed tomography sequence image segmentation results affects the diagnosis and treatment effect. Therefore, it has important value. In the traditional manual segmentation process, segmentation has poor repeatability, time-consuming, and accuracy cannot be guaranteed. This paper proposes a computed tomography sequence image edge segmentation optimization algorithm using improved convolution neural network. The images that meet the requirements are divided into different types, and the edge information of the real image is extracted as much as possible. Edge information extraction and de-noising of computed tomography sequence images. Through the de-convolution layer and other network layers, the details and corresponding space of the target can be gradually recovered, so as to effectively maintain the resolution of the computed tomography sequence image and complete the optimal edge segmentation of the computed tomography sequence image. The results show that the overall performance of the proposed method is better, and it has certain reference value for related research in the field of image segmentation. However, this paper has done a lot of work in reducing algorithm complexity, there are still many shortcomings in large-scale computed tomography sequence image segmentation, and there is a lack of fast computed tomography sequence image segmentation algorithm. Therefore, in future works, we should focus on a fast segmentation method of large-scale computed tomography sequence image to improve segmentation efficiency.
